# Medicina Nautica

**Published:** 1816-02

**Authors:** 


### MEDICINA NAUTICA.

*To the Editors of the Medico-Chirurgical Journal & Review.*

GENTLEMEN,

PERMIT me to enclose you the following Cases and Remarks for your department of "Naval Medicine," to which I shall be always happy to contribute as occasion may offer. Although I withold my name from the public, you can vouch for the authenticity of the enclosed papers, and that the remarks are the result of not very limited experience.

With every wish for the success of your very laudable undertaking in the Naval Medical department, and also for the prosperity of your valuable Journal,

I am, &c.

B.*

### *WEST INDIA FEVER.*

On the 4th of November, 1814, we arrived at Barbadoes, where we continued until the 12th; during which time several cases of Fever presented amongst our people,

---

* We are personally acquainted with the writer, and know him to be a Gentleman of talents and very great experience as a Staff Surgeon. No communications can ever be more acceptable to us than those which we receive from B.—EDITORS.

probably from their indulging in intemperance and exposure to solar influence, whilst employed on shore, watering, and performing other necessary duties of the ship, which may reasonably be considered as the exciting causes of the disease. The following is a case exemplifying the most prominent symptoms of the disease, and the means of cure adopted.

Nov. 9th, 1814. Robert Jenkins, seaman, ætat. 20, of low stature and plethoric habit of body, was admitted on the sick list. Countenance anxious, and expressive of much distress; face flushed; eyes suffused and watery.

Complained at seven, P. M. of having been afflicted with slight shiverings, succeeded by universal lassitude all over the body and limbs; head-ache, chiefly referred to the forehead and bottom of the orbits, with occasional lancinating pains shooting towards the temples, and great intolerance of light; pulse 110, quick and tense; skin dry; temperature 101° Fahr.; tongue white and coated, with red edges; thirst great; bowels constipated. Quamprimum detrahatur sanguis e arteria temporali ad ℥xl.

℞. Hydr. submuriat. gr. x; pulv. jallap. ℈j; confect. aromat. q. s. fiat bolus statim sumendus. Hauriatur decoct. hordei pro potu communi.

10th. Eight, A. M, expressed much relief after the bleeding, and has had three large, black, fœtid evacuations since morning. Countenance still very anxious; breathing oppressed; head-ache and intolerance of light distress him much; pulse 110 and wiry; skin dry; temperature 100°; tongue moist and coated; thirst insatiable. Detrahantur sanguinis e temp. art. ℥xxx. Continuetur potus; tondeatur caput, et lavationes frigidæ capiti constanter applicandæ sunt.

℞. Hydrarg. submuriat. gr. vj; pulv. antimonial. gr. ij; cons. rosar. q. s. fiat bolus, quartis horis sumendus.

Seven, P. M. expresses himself much easier; countenance not so anxious; breathing not so laborious; head-ache still continues a little; pulse 112, soft and compressible; skin dry; temperature 101°; thirst great; bowels not moved. Applicentur cucurbitulæ cum ferro temporibus. Continuentur bolus, et applicationes frigidæ capiti.

11th. Eight, A. M. about eight ounces of blood were obtained from the temples; has had a tolerable night; countenance cheerful; breathing easy; head ache gone, and he complains of little inconvenience from the light; pulse 90 and soft; tongue coated and moist; temperature 98°; thirst abates; bowels moved twice; stools large, black, and fœtid. Continuentur potus, et bolus sub. hyd. c. pulv. ant. ut heri.

Seven, P. M. continues better.

12th. Slept well during the night; countenance lively; breathing natural; no head-ache; pulse 75, and soft; skin moist; temperature 98°; tongue moist and white; thirst moderate; has had three copious evacuations during the night, the last of a bright yellow colour, and he now complains of his gums being a little sore. Continuetur bolus mane nocteque.

13th. A good night, and expresses himself free from complaint; mouth a little affected from his medicines; pulse 68, and soft; tongue white, but not coated; thirst moderate; bowels easy; stools yellow and copious. Omittatur bolus hydrarg. submuriat. cum pulv. antimonial. Bibat decoctum hordei ad libitum.

14th. Slept well during the night, and now complains only of his mouth being a little sore; pulse and skin pretty natural; tongue moist; thirst moderate; bowels moved twice; stools of pretty natural colour and consistence. Continuetur potus.

15th to 20th. Continued progressively amending; strength and appetite rapidly improving. Habeat vini rubri ℔j in die.

30th. Discharged cured.

REMARKS.

I have found the above method of treatment, with little variation, to succeed in effecting a cure in the fever cases that came under my care whilst at Barbadoes, and likewise some that occurred subsequently; and although mercury is objected to by many practitioners of acknowledged abilities and experience, I have generally found, after premising the necessary evacuations, that mercury had the happiest effects when judiciously administered, namely, in re-establishing all the healthy secretions and excretions.

---

### Case of Hepatic Affection.

April 6th, 1815. Mr. R. E. N. ætat. 35, slender make and spare habit of body; countenance sallow; complained of being afflicted with a severe settled pain at the pit of the stomach, urging to vomit; loss of appetite, and restless nights, with lassitude all over the body and limbs. Pulse soft and rather full; skin pretty natural; tongue a little furred and yellow; bowels constipated; thirst moderate. Mittatur sanguis e venâ brachii ad ℥xvj.

℞. Ext. colocynth. comp. gr. xv; hydrarg. submuriat. gr. vj. M. fiat bolus, statim sumendus.

7th. Has had two copious evacuations of a light clay colour; pain at pit of stomach still severe, and vomits almost every thing he takes: pulse not so full, and tolerably regular; skin pretty natural; tongue furred; thirst moderate; no inclination for food.

℞. Hydrarg. submuriat. gr. xv. Ext. opii gr. ß. confect. aromat. q. s. fiat bolus, mane, meridie, nocteque sumendus, et capiat haustum effervescentem horis quartis.

8th. Has had a restless night; pain at pit of stomach continues; urging to vomit somewhat abated; pulse soft; skin natural; tongue yellow; thirsty; bowels not moved. Continuetur haustus et bolus hydrarg. submuriat. sine opio. Bibat decoctum hordei ad libitum.

9th. A restless night; pain at pit of stomach much as before; has now little or no vomiting; pulse, skin, and tongue as yesterday; bowels moved freely; stools dark and fœtid. Continuetur bolus e hydrarg. submuriat.; omittatur haustus effervescens.

10th. Pain at pit of stomach entirely removed; mouth affected by his medicine, with considerable flow of saliva; pulse a little quick; skin natural; tongue not so yellow; thirsty; bowels easy; stools large, and of a bright yellow colour; and has some appetite for food. Omittatur bolus e hydrarg. submuriat.

℞. Aluminis sulphatis ℈j.; aq. puræ ℥viii.; mel acetat. ℥i; M. fiat gargarisma pro ore, sæpe utend.

11th. A tolerable night, and expresses himself free from complaint, only his mouth very sore, with great flow of saliva. Continuetur gargarisma pro ore. Bibat decoctum hordei ad libitum.

12th. No complaint but soreness of mouth and debility; bowels regular; stools large, and of pretty natural colour and consistence. Continuetur gargarisma. Habeat vini rubri ℔ß in die.; arrow root, sago, &c. pro re nata.

From this period he continued daily improving, requiring only an occasional laxative, and a continuance of the gargle, and his health is now completely re-established.

30th. Discharged cured.

REMARKS.

This patient stated that he had been for some weeks afflicted with a constant settled pain at pit of the stomach, which progressively increased to the period at which he made application (6th of April); and under which complaint he was sensibly declining. Venæsection, blistering, and fomentations had been used, with occasional laxatives, without producing any alleviation whatever. He was much averse to taking mercury; however, upon pointing out to him his situation, and the absolute necessity of exhibiting mercury, he willingly consented; the effect of which was, as will be perceived by the preceding case, that as soon as the system became sensibly affected, the disease totally disappeared, and he now expresses himself in better state of health than he has been for some years.

---

### Case of Dysentery.

In the months of January and February, 1815, we were employed in the Gulf of Mexico, where our people were much exposed to fatigue and cold, whilst away in boats, performing subservient duties to the army, which was at that time encamped before New Orleans; and also from the weather about this period proving unsettled, with considerable decrease of atmospherical temperature, dysentery began to make its appearance, which continued to attack individuals for some time afterwards, varying in its degrees of severity according as the constitution was more or less susceptible of its attack. I conceived it might not be useless to subjoin the following case, as illustrative of the most prominent symptoms and method of treatment usually adopted in this most harassing malady.

February 12, 1815. George Hartfield, seaman, ætatis 21, of stout make and plethoric habit of body; countenance dejected, eyes languid; complained of head-ache, nausea, and urging to vomit, accompanied with severe tormina and frequent desire to stool, without being able to evacuate any thing but a little bloody, fæculent matter. Pulse quick and rather full; skin dry and hot; tongue white and clammy; thirst much: says that these complaints were ushered in by slight shiverings, with sense of cold all over the body and limbs. Quamprimum mittatur sanguis e venâ brachii ad ℥xxx.

℞. Hydrarg. submuriat. ℈j; ext. opii gr. j; confect. aromat. q. s. fiat bolus statim sumendus. Bibat decoctum hordei pro potu communi.

13th. Eight, A. M. restless night; countenance very anxious, and expressive of great distress: tormina, with desire to stool, continue without passing any thing but a little blood, and he has occasionally had urging to vomit; pulse quick; skin dry and warm; tongue white, and he is thirsty. Habeat balneum calidum statim, et postea capiat olei Ricini ℥jß. aq. menth. pip. ℥j. M. fiat haustus. Seven, P. M. experienced much relief from the bath; tormina easier, and he has had two dark-coloured grumous evacuations since morning; pulse quick; skin warm; tongue white; thirst much; and has occasionally desire to vomit. Repetantur bolus et balneum calidum.

14th. Eight, A. M. slept a little during the night; tormina and desire to stool considerably alleviated; no urging to vomit; countenance more lively; pulse quick; skin warm, and a little moist; tongue white; thirsty. Continuatur bolus e hydrarg. c. opio.—Vespere. Occasionally tormina and tenesmus during the day, with small fæculent bloody stools. Pulse and other functions much as morning. Repetatur bolus e hydrarg. submuriat. c. opiô.

15th. Eight, A. M. a better night; tormina and tenesmus considerably alleviated; stools fæculent and mixed with blood. Repetatur bolus e hydrarg. submuriat. sine opio.—Vespere. Has had two large fæculent evacuations during the afternoon, and expresses himself now very easy. Repetatur bolus e hydrarg. submuriat.

16th. Eight, A. M. slept well; complains little of tormina or tenesmus, but says his mouth is very sore; has had one large evacuation of a bright yellow colour since morning; pulse a little quick; skin moist; tongue white; thirst moderate.

℞. Hydrarg. submuriat. gr. v; ext. opii gr. ß; pulv. antimonial. gr. iij; confect. aromat. q. s. fiat bolus, mane nocteque sumendus.

℞. Aluminis sulphatis ℈j aq. puræ ℥viij. M. fiat gargarisma pro ore, sæpe utend.

17th. Eight, A. M. continues perfectly easy; stools large and of a dark yellow colour; mouth very sore, and he spits considerably. Omittatur bolus et continuetur gargarisma.

18th. Eight, A. M. complains of nothing, except his mouth; pulse and other functions pretty regular. Continuetur gargarisma pro ore. Habeat vini rubri ℔ß in die.

19th. Eight, A. M complains only of his mouth being sore; stools of natural colour and consistence. Continuentur remedia.

20th to 30th. Has continued getting better, and is now regaining his strength, appetite, &c. rapidly. Omittantur omnia.

REMARKS.

I had long been in the habit of administering mercury in dysentery, either by itself, or combined with antimonial powder and opium, according to existing circumstances; but I must observe, that I seldom gave it in such large doses till Mr. Johnson's valuable publication on the "*Influence of Tropical Climates,*" fell into my hands; since which, a field for considerable experience in that disease presented, whilst surgeon to a naval establishment on a foreign station, and subsequently in accompanying our expedition to New Orleans; and I am gratified to be able to state, that by the early abstraction of blood from the system, when excitement indicated it to be necessary, with the subsequent administration of calomel, either by itself or combined with opium and antimonial powder, in the manner above related, according to existing circumstances, until its specific effects became visible, every symptom of dysentery invariably disappeared: in short, in no one instance of recent dysentery has this mode of practice failed. Sometimes the warm bath became necessary, when the irritation of stomach and vomiting were distressing, accompanied with a dry, constricted state of the skin. And what still more confirms the utility of this practice is, that not an individual case of relapse has occurred, and the major part of those who were afflicted with the malady, express themselves in better health than previous to the attack of disease; it is true, several experienced considerable annoyance from mercurial ptyalism existing for some time; however, the temporary inconvenience and debility produced are by no means to be compared with that arising from protracted disease and subsequent disorganization, which I have frequently observed to be no uncommon occurrence in dysentery differently treated.

During convalescence, nothing appeared necessary but attention to the state of bowels, with light generous diet, a moderate allowance of wine, and occasionally some vegetable tonic bitter.

I am aware of the discrepancy of opinion which exists, relative to the treatment of dysentery; however, it is not my intention to enter into any theoretical disquisitions, but to confine myself strictly to practical facts, obtained from clinical observation. B.

